# Ventrolateral orbital cortex as a functional hub in chronic pain and comorbid depression

**DOI:** 10.3389/fnins.2026.1745445

**Published:** 2026-01-30

**Authors:** Hai-Yan Sheng, Hui-Na Song, Meng-Xin Hu, Ru-Ling Shi, Lin-Hua Jiang

**Affiliations:** 1 Sino-UK Joint Laboratory of Brain Function and Injury of Henan Province, and Department of Physiology and Pathophysiology, School of Basic Medical Sciences, Xinxiang Medical University, Xinxiang, China; 2 Henan Key Laboratory of Neurorestoratology and Protein Modification, Henan Medical University, Xinxiang, China; 3 School of Public Heath, Xinxiang Medical University, Xinxiang, China; 4 School of Biomedical Sciences, Faculty of Biological Sciences, University of Leeds, Leeds, United Kingdom

**Keywords:** 5HT, 5HT receptors, chronic pain, comorbidity, depression, VLO

Depression is well-known for its comorbidity with chronic pain in humans. Chronic pain was initially proposed as a variant of depressive disease (Blumer and Heilbronn, 1982), which elicited intense debate surrounding the causality of these two conditions. Subsequent epidemiological studies show that chronic pain predisposes individuals to experience depression (Magni et al., 1993) and patients with depression exhibit increased incidence of pain (Von Korff et al., 1993). It has been nowadays recognized that chronic pain and depression are comorbid, being bidirectional (Bair et al., 2003; Johnston and Huckins, 2023). As revealed by functional magnetic resonance imaging, the functional connectivity between the central amygdala (CeA) and dorsal raphe nucleus (DRN) is reduced in patients with comorbidity of chronic pain and depression, but not in chronic pain patients without depression (Zhou et al., 2019). Moreover, activation-likelihood estimation meta-analyses suggest that pain with concomitant depression is associated with the right amygdala, whereas depression with concomitant pain is primarily related to the left dorsolateral prefrontal cortex (Zheng et al., 2022). Collectively, accumulating evidence suggests the comorbidity of chronic pain and depression exacerbates each other, which imposes significant challenges in treating these conditions. Elucidating the neuronal mechanisms underpinning the comorbidity of chronic pain and depression can facilitate identification of new targets and development of better or more effective therapeutics. Recent studies from us and other groups provide experimental evidence to suggest that the neuronal activity of ventrolateral orbital cortex (VLO) plays an important role in transducing signals mediating chronic pain and comorbid depression, and emerges as a promising therapeutic target for chronic pain and negative emotions arising from chronic pain.

## Anatomy and cytoarchitectonic of VLO

Orbitofrontal cortex (OFC) is located on the ventral surface of the most anterior part of the cerebral frontal lobe, directly covering the dorsosuperior aspect of the orbital bony structure, one of the three major functional subdivisions of prefrontal cortex (PFC) (Wilson et al., 2010), and interacts most closely with the limbic system (Yang et al., 2025). Posteriorly, it is adjacent to the rostral segment of anterior cingulate cortex (ACC) and, laterally, bounded by the sulcus olfactorius and connects with the anterior edge of the agranular insular cortex. OFC in rats are subdivided into five distinct subregions, namely, medial orbital cortex, ventral orbital cortex, VLO, lateral orbital cortex and dorsolateral orbital cortex (Vertes et al., 2023). OFC in mice is often separated into three primary functional subregions, namely, medial orbital cortex, VLO and lateral orbital cortex, based on the specificity of functional connectivity and virus tracing results (Zhang et al., 2024). Multiple projections from different sensory modalities, including olfaction, gustation, vision, audition, and somato-sensation, are convergent on OFC, which serves as a hub for receiving and integrating various sensory inputs (Zald and Kim, 1996a). In addition, OFC is a core area for emotion processing, due to its unique capacity of decoding reward and punishment signals and converting them into emotional experience (Jessup and O'Doherty, 2014) and reducing the activity of amygdala to control negative emotions such as fear (Zald and Kim, 1996b), and is critically involved in the generation of emotions, such as pleasure, regret, embarrassment, anger and sadness. Moreover, OFC holds a cognitive map, an essential component that enhances the ability for prediction and inference and, in turn, guides decision-making processes underlying adaptive behaviors (Ma et al., 2025).

VLO accounts for the major portion of OFC and is classified as a dysgranular cortex, devoid of a distinct granular cell layer. The cytoarchitectonic of VLO is similar in mice and rats, composed of five layers (I, II, III, V and VI) (Van De Werd and Uylings, 2008; Van De Werd et al., 2010). Layer II, also known as the external pyramidal layer, is predominantly composed of small pyramidal neurons that receive afferent signals from layer I and form close connections with adjacent cells via local circuits, functioning primarily in the integration of local information. Layer III, or the middle pyramidal layer, has a larger volume, a higher distribution density and more active inter-regional projections, with pyramidal neurons being the major neuron type. Layer V, or the internal pyramidal layer, is the core output layer of the VLO, harboring the highest density of pain regulation-related projection neurons, and the vast majority of them are large- diameter excitatory pyramidal neurons (Babalian et al., 2019). Parvalbumin-positive interneurons are more densely distributed in layer V, and integrates local regulatory functions with thalamic feedback capabilities (Vertes et al., 2023). The neuron population in layer VI, or the multiform layer, exhibits high heterogeneity, and is comprised of spindle and small pyramidal neurons. The density of parvalbumin-positive interneurons in layer VI is intermediate between that in layers II and III and that in layer V, and serve to maintain the excitation-inhibition balance of the local circuits.

## VLO in chronic pain

An earlier study based on morphological characterizations has identified that VLO primarily receives projections from ipsilateral thalamic nucleus submedius (Sm) and sends efferent nerve fibers to the periaqueductal gray (PAG) in the midbrain, forming the Sm-VLO-PAG pathway as the descending analgesic pathway (Coffield et al., 1992). VLO is a core component of this descending pathway and has attracted considerable research attentions for its role in transducing pain signals. Studies, using rodents to assess the radiant heat-induced tail flick reflex, have identified a range of neuromodulators and neurotransmitters, including opioids, glutamate, gamma-aminobutyric acid (GABA), 5-hydroxytryptamine (5-HT) and dopamine, and their respective cognate receptors as critical mediators of VLO-driving analgesic effects (Tang et al., 2009). The key mechanism underpinning such analgesic effects is that activation of the descending pathway inhibits the transmission of nociceptive information at the spinal cord level and thereby blocks transduction of pain signals further to the brain.

In mice, neuropathic pain, evoked by chronic constriction injury of infraorbital nerve-induced trigeminal neuralgia (TN) and spared nerve injury-induced sciatica, resulted in decreased activity of excitatory neurons, accompanied by increased activity of inhibitory interneurons within the VLO (Sheng et al., 2020; Huang et al., 2021). As shown in a recent study, astrocytes in the VLO in the TN rats were highly activated and, importantly, inhibition of such astrocyte activation sufficiently restored the activity of glutamatergic neurons and produced analgesia (Islam et al., 2025).

It is worth mentioning the recent study showing that itch-specific neurons are also localized within the VLO, but their functional activity and projections appear distinct (Jiang et al., 2022). Pain-specific and itch-specific neurons constitutes two entirely non-overlapping neuronal subpopulations. Pain-specific neurons are projected to the agranular insular cortex, while itch-specific neurons are mainly projected to the piriform cortex (Jiang et al., 2022). These findings provide evidence for the pluralistic role of the VLO as a sensory integration center in addition to pain perception.

## VLO in depression comorbid with chronic pain

**Figure 1:**
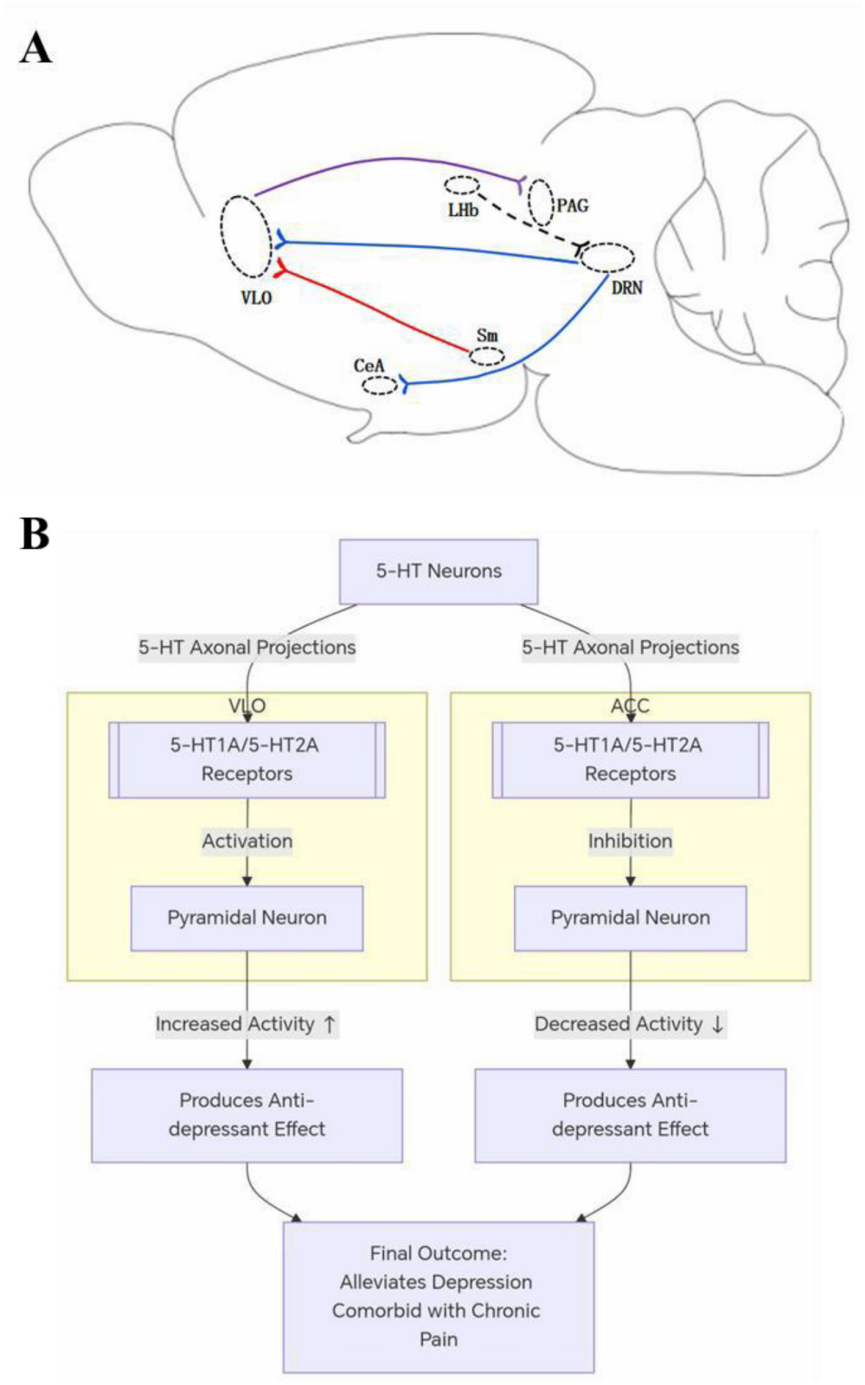
Schematic illustration of the VLO in the neural pathways in chronic pain and comorbid depression. **(A)** Activation of the pathway in purple results in an analgesic effect, activation of the pathways in blue leads to alleviation of chronic pain-induced depressive-like behaviors, and activation of the pathway in red results in both analgesic and anti-depressant effects. The dotted line represents the pathway, by which chronic pain leads to a decrease in the activity of 5-HTergic neurons in the DRN. **(B)** The anti-depression mechanisms mediated by 5-HT1A and 5-HT2A receptors in the VLO and ACC. ACC, anterior cingulate cortex; CeA, central amygdala; DRN, dorsal raphe nucleus; LHb, lateral habenular nucleus; PAG, periaqueductal gray; Sm, nucleus submedius; VLO, ventrolateral orbitofrontal cortex.

Neuropathic pain induced salient depressive-like behaviors in rodents (Guo et al., 2018). As described above for the Sm-VLO-PAG pathway as an analgesic pathway, selective activation of the Sm-VLO constituent alleviated depressive-like behaviors and, in contrast, activation of the VLO-PAG constituent failed to elicit significant anti-depressant effect (Sheng et al., 2020), revealing that the activity of the Sm-VLO constituent is of particular importance in chronic pain-induced depression (Figure 1A). Our early study showed that activation of the excitatory glutamatergic neurons or suppression of the inhibitory GABAergic neurons in the VLO was sufficient to alleviate chronic pain-induced depressive-like behaviors in the TN mice (Sheng et al., 2020), indicating the neuronal activity of VLO is critical in determining chronic pain-related depression (Figure 1A).

The activity of 5-HTergic neurons within DRN, one of the major sources of 5-HT, has been shown to be reduced in mice with depressive-like behaviors (Zhou et al., 2019, 2022; Zhao et al., 2024). Our recent study has revealed that the activity of 5-HTergic neurons in the DRN was also decreased in mice with TN-induced depressive-like behaviors (Sheng et al., 2025). In addition, our recent study has demonstrated that DRN sends direct neural projections into the VLO and a majority of such neural projections are 5-HTergic. Importantly, both anterograde and retrograde activation of the DRN-VLO pathway consistently exerted anti-depressant effects in the TN mice (Sheng et al., 2025). These results indicate that the DRN-VLO pathway act as a neural pathway in transducing chronic pain-induced depression (Figure 1A). It is important to point out that the CeA, a core output nucleus of the amygdala complex, serves as a pivotal integrating station in the brain for emotional processing. CeA also functions as a key brain region mediating transformation of pain from sensory discrimination, for example, pain location or intensity, to emotional experiences such as aversiveness, anxiety or depression associated with pain. An early study provides evidence that 5-HTergic neurons in the DRN project into the somatostatin (SOM)-expressing interneurons in the CeA and activation of the DRN-CeA^SOM^ pathway reduced depressive-like behaviors in mice with spared nerve injury-induced neuropathic pain or complete Freund's adjuvant-induced inflammatory pain (Zhou et al., 2019). Therefore, the DRN-VLO pathway and the DRN-CeA pathway act as parallel neural pathways in transducing chronic pain-induced depressive-like behaviors (Figure 1A).

A recent study has reported that sequential input from the lateral habenula (LHb) to the DRN induces two spatially different responses through distinct signaling mechanisms in 5-HTergic neurons of the DRN, namely, a transient, single-synapse excitation mediated by glutamatergic signaling followed by sustained inhibition via the 5-HT1A receptor (Figure 1A) (Lynn et al., 2025). In other words, in a state of depression, LHb becomes hyperactivated and inhibit the 5-HTergic neuronal activity in the DRN (Liu et al., 2024; Lynn et al., 2025).

## 5 -HT synaptic mechanisms of VLO in depression comorbid with chronic pain

Our recent study has shed light on the role of synaptic mechanisms mediated by release of 5- HT and activation of distinct 5-HT receptor subtypes in the anti-depressant effects produced by activation of the DRN-VLO pathway in the TN mice. Activation of the DRN-VLO pathway promotes 5-HT release in the VLO. 5-HT in turn stimulates the 5-HT2A receptors on the excitatory glutaminergic neurons and, in addition, the 5-HT1A receptors on the inhibitory GABAergic interneurons via a disinhibition mechanism, and these two distinct mechanisms work together to enhance the excitatory output activity of pyramidal neurons in the VLO (Sheng et al., 2025). ACC has been well established to play a critical role in mediating pain-related aversion by studies using different pain models (Journée et al., 2023). Of interest, a recent study has shown that activation of the 5-HT1A and 5-HT2A receptors in the ACC inhibits rather than stimulates the activity of pyramidal neurons, producing both analgesic and anti-depressant effects in mouse models (Hammo et al., 2025). Therefore, evidence from recent studies suggests that stimulation of 5-HT release from axon terminals and activation of the 5-HT1A and 5-HT2A receptors enable dampening depression comorbid with chronic pain, either by activating the activity of pyramidal neurons in the VLO (Sheng et al., 2025) or inhibiting the activity of pyramidal neurons in the ACC (Hammo et al., 2025) (Figure 1B). Conceivably, enhancing 5-HT synaptic transmission in the frontal cortex is a promising strategy for alleviating chronic pain-induced depression. However, it is worth pointing out that compared to ACC in rodents, ACC in humans appears to be structurally and functionally more complex, with different subregions responsible for distinct pain-related processes. For instance, the subgenual subregion is activated during experiences of negative emotions, and the pregenual subregion is activated in response to happy emotion and mediates pain responses (Vogt, 2005). Therefore, due considerations of such differences between humans and rodents are required, when translating the findings from preclinical studies using rodent models into clinical interventions.

In summary, VLO, a critical component of OFC, serves not merely a passive participant in the endogenous analgesic system but also emerges as an active, multifunctional hub in regulating the negative emotions, particularly depression, induced by chronic pain. Further investigations are required to define the dynamic changes accompanying the transition from acute to chronic pain and, specifically, the balance of excitatory and inhibitory neuronal activities and the roles of various neurotransmitters and their cognate receptors within the VLO, providing in-depth insights into the core neural mechanisms of chronic pain maintenance and depression induction. Such information is important for exploring the perspective on targeting such neural mechanisms to treat depression arising from chronic pain.
